# Association between IGF-1 levels and MDD: a case-control and meta-analysis

**DOI:** 10.3389/fpsyt.2024.1396938

**Published:** 2024-06-11

**Authors:** Xin Qiao, Jiaxin Yan, Zongjun Zang, Lei Xi, Wenli Zhu, En Zhang, Lijuan Wu

**Affiliations:** ^1^ School of Humanities and Management, Wannan Medical College, Wuhu, China; ^2^ Department of Psychiatry, Wuhu Fourth People’s Hospital, Wuhu, China; ^3^ Department of Psychiatry, Shenyang Mental Health Center, Shenyang, China

**Keywords:** IGF-1, depressive disorder, first-episode and drug-naïve, case-control, meta-analysis

## Abstract

**Purpose:**

Insulin-like growth factor-1 (IGF-1) has a variety of neurotrophic effects, including neurogenesis, remyelination and synaptogenesis, and is an effective regulator of neuronal plasticity. Although multiple studies have investigated IGF-1 in depression-related disorders, few studies have focused on patients with a first episode of clearly diagnosed depression who had never used antidepressants before. Therefore, this study investigated first-episode and drug-naïve patients with depression to supplement the current evidence around IGF-1 levels in depressive disorders.

**Patients and methods:**

This study consisted of two parts. In the first part, 60 patients with first-episode and drug-naïve depression and 60 controls matched for age, sex, and BMI were recruited from the outpatient department of the Fourth Hospital of Wuhu City, and the community. The case-control method was used to compare differences in serum IGF-1 levels between the two groups. In the second part, 13 case-control studies were screened through the database for meta-analysis to verify the reliability of the results.

**Results:**

Results of the case-control study demonstrated that serum IGF-1 levels are significantly higher in patients with first-episode and drug-naïve depression compared to healthy controls (*p*<0.05), although there was no significant difference between men and women with diagnosed MDD, there was no significant correlation between serum IGF-1 level and age in patients with depression and no significant correlation between IGF-1 level and the severity of depression. The meta-analysis corroborates these findings and demonstrated that IGF-1 levels are significantly higher in MDD patients than in healthy controls.

**Conclusion:**

Patients with first-episode and drug-naïve depression have higher IGF-1 levels, but the exclusion of confounding factors in studies of IGF-1 as it relates to depressive disorders must be taken into consideration strictly, and additional research is needed to fully understand the critical role of IGF-1 in depression.

**Systematic review registration:**

PROSPERO, identifier CRD42023482222.

## 1 Introduction

Although depressive disorder is a common mental disorder, current diagnostic and treatment methods rely on the subjective identification of clinical symptoms by psychiatrists (1), and its pathogenesis remains unknown. Effective peripheral biomarkers will greatly improve the diagnostic accuracy of depressive disorder.

In recent years, neurotrophic factors including brain-derived neurotrophic factor (BDNF), vascular endothelial growth factor (VEGF), nerve growth factor (NGF), glial cell-line derived neurotrophic factor (GDNF), and insulin-like growth factor (IGF-1) have been widely explored in depression-related studies (2–5). IGF-1 plays a critical role in the life cycle of a cell and performs numerous cellular functions including growth regulation, survival and differentiation. IGF-1 also has neurotrophic and neuroprotective effects, and is essential to enhancing the survival and plasticity of neurons in the brain, many of which influence cellular neuroplasticity of the central nervous system (6, 7). IGF-1 has been shown to contribute to neurogenesis, myelination, remyelination, neuromodulation, and synaptogenesis – each processes that are impaired in affective disorders (8). Furthermore, IGF-1 is a negative mediator of apoptosis following brain injury and acts through an endogenous neuroprotection system (9). A new large-sample study shows a strong link between high and low IGF-1 concentrations and brain disease, highlighting the potential of IGF-1 as a biomarker for stratifying brain health risks (10). It has also been suggested that decreased IGF-1 expression in the brain disrupts neuroplasticity mechanisms and promotes inflammatory pathways, leading to morphological deterioration in brain regions responsible for emotional and cognitive processing. Hence, abnormal IGF-1 activity may be associated with the development of mood disorders (7, 11, 12). To date, the results of studies on IGF-1 levels in patients with depression remain controversial, and few studies have focused on patients with a first episode of clearly diagnosed depression who had never used antidepressants before. Whether there are different trends in IGF-1 levels in these patients is a matter of interest. Therefore, this study investigated first-episode and drug-naïve patients with depression to supplement the current evidence around IGF-1 levels in depressive disorders. Here, we performed a unique case-control study and subsequently verified the reliability of the results through meta-analysis in an effort to update the evidence of clinical application of IGF-1 in patients with depression, and to aid the field in providing a reliable basis for clinical evaluation and nursing of patients with depression.

## 2 Materials and methods

### 2.1 Participants

Sixty patients with first-episode and drug-naïve depression and 60 healthy volunteers were recruited from the outpatient department of the Fourth Hospital of Wuhu City, and the community, respectively, between December 2021 and August 2023 and were enrolled to participate in the study. The healthy controls were matched for age, sex and BMI, and participants with serious physical diseases were excluded.

Inclusion criteria for MDD patients were (1) aged 18–65 years; (2) depression diagnosed according to international Classification of diseases (ICD-10); (3) first-episode and drug-naïve depression. Exclusion criteria were (1) mental disorders due to organic lesions or physical diseases; (2) mental disorders due to drugs or active substances; (3) unable to complete the test or refusal to participate.

This study was approved by the Ethics Committee of the Fourth People’s Hospital of Wuhu. Prior to participation in the study, all participants provided oral and written informed consent.

### 2.2 Blood tests and clinical assessments

Fasting venous blood (5 mL) was collected within 2 days after enrollment and placed in an anticoagulant test tube at room temperature for 1 h. The supernatant was centrifuged at 3030 xg for 10 min, then placed in a refrigerator at −80°C until testing. The enzyme-linked immunosorbent assay (ELISA) kit (sCD36, Enzyme-linked Biology, China) for IGF-1 was used to detect the level of neurotrophic factors in serum according to manufacturer instructions. Details about the ELISA analysis as follows: Assay range: 5 ng/mL–160 ng/mL. Standard concentration was followed by: 160, 80, 40, 20, 10, 5 ng/mL. Sensitivity: The minimum detectable dose of Human IGF-1 is typically less than 1.0 ng/mL. Reproducibility: Intra-assay CV(%) is less than 10% and Inter-assay CV(%) is less than 15%. Read the Optical Density (O.D.) at 450 nm using a microtiter plate reader (Thermo Fisher Scientific, USA). And 3 replicates each sample was tested in.

The Hamilton Depression Rating Scale (HAMD) was used to assess the severity of symptoms in patients with depression, the HAMD consists of 17 items, with higher scores indicating greater severity of depressive symptoms. According to the HAMD scores, the patients were divided into four groups: no depression score ≤7, mild depression score 7 < total score ≤17, moderate depression score 17 < total score ≤24, and severe depression score > 24.

### 2.3 Literature search

The study was conducted in accordance with the PRISMA (Preferred Reporting Items for Systematic Reviews and Meta-analyses) 2020 statement and was registered with PROSPERO (No. CRD42023482222). The PRISMA 2020 checklist is provided in **Supplementary Table 1**.

A systematic literature search of PubMed, Embase, and Web of Science was conducted up to October 2023, using thesaurus terms from each database in a complete search strategy for each keyword. The basic components of the search terms included: (1) “depressive disorder” and synonyms; (2) “IGF-1” and synonyms. Specific search strategies are shown in **Supplementary Table 2**.

### 2.4 Identification of eligible studies

The inclusion criteria were as follows: (1) The study design was case-control study, cohort study or cross-sectional study including a depression group and a healthy control group; (2) All patients were diagnosed with depression; (3) IGF-1 concentrations in serum and/or plasma were reported.

Exclusion criteria were as follows: (1) studies on patients with other mental disorders (e.g., schizophrenia, bipolar depression, Alzheimer’s disease, unless separate data were available for individuals with unipolar depression); (2) depression due to other physical diseases (such as stroke, traumatic brain injury, perinatal depression or cancer); (3) reviews, letters, editorial comments, case reports, conference abstracts, unpublished articles and non-English articles.

### 2.5 Data extraction

Data were extracted by two investigators independently and included lead author, publication year, sample source, sample size, participant characteristics (race, sex, age, height, weight), depression rating instrument, antidepressant use at enrollment, and IGF-1 level (mean and standard deviation). When data in a study were missing or not reported, the corresponding authors were contacted to obtain complete data, when available.

### 2.6 Quality assessment

The Newcastle Ottawa Scale (NOS) was used to assess the quality of included studies depending on three main factors: selection, comparability, and exposure, each of which contained subordinate items. The total score for each study ranged from 0 to 9, and studies with a score of 7 to 9 were considered high quality. Two investigators independently assessed the quality and level of evidence for the eligible studies, and any discrepancies were resolved through discussion.

### 2.7 Statistical analysis

Data normality was assessed using descriptive skewness and kurtosis methods, Q–Q plots and Kolmogorov-Smirnov assumption test (**Supplementary 3**). Categorical data were analyzed using chi-square tests, the *t* test was used for measurement data. Pearson correlation analysis was used for correlation analysis. The above data were analyzed by SPSS21.0.

Evidence synthesis was performed in Review Manager version 5.4 (Cochrane Collaboration, Oxford, UK). Standard mean differences (SMDs) and 95% confidence intervals (CIs) were used to assess effect sizes (ES) between the two groups. SMD values greater than 0 indicated that IGF-1 level is higher in depressed patients than in healthy controls. The heterogeneity of the studies was assessed by the chi-square (χ^2^) test and the heterogeneity index (I^2^) (13). The I^2^ statistic was used to estimate heterogeneity between studies: χ^2^ *p*< 0.05 or I^2^>50% heterogeneity was considered significant. When significant heterogeneity was detected, a random effects model was used to calculate its ES and a Z-test was used to determine the significance of the pooled SMD. Absolute values of 0.2, 0.5, and 0.8 for SMD indicated low, medium, and high ES, respectively (14). In addition, a sensitivity analysis was performed to assess the impact of included studies on outcomes with significant heterogeneity. The results of the included studies were tested using Stata version 17.0 (Stata Corp, College Station, TX, USA), and *p* < 0.05 was considered statistically significant for publication bias. Meta-regression and subgroup analyses were used to explore sources of heterogeneity according to the following potential confounders: mean age, sex distribution (proportion of females), race, sample source (serum/plasma), antidepressant use(single/mixed/no) and study quality.

## 3 Results

### 3.1 Results of the case-control

**Table 1** shows the clinical characteristics of all participating investigators: There were no significant differences in age, sex, or BMI between the two groups. There was a significant difference in IGF-1 levels between the two groups, and serum IGF-1 levels were significantly increased in patients with first-episode and drug-naïve depression (MDD:149.81 ± 34.35 ng/ml, control:128.23 ± 25.83 ng/ml, *p*<0.05). However, there was no significant difference in IGF-1 level between men and women MDD patients (*z*=-0.633; *p*=0.531). Pearson correlation analysis showed that there was no significant correlation between serum IGF-1 level and age in patients with depression (r=0.103;*p*=0.434), and there was no significant correlation between serum IGF-1 level and the severity of depression (r=-0.271; *p*=0.096).

**Table 1:** Comparative profiles of the MDD patients and healthy controls.

Variables	MDD	Control	*t*-value	P-value
Number	60	60	–	–
Age(year)*	35.48 ± 11.77	34.63 ± 14.05	0.359	0.720
Male/female	23/37	21/39	0.144	0.705
BMI (kg/m^2^)*	23.08 ± 3.37	22.13 ± 3.98	1.423	0.157
IGF-1(ng/ml)*	149.81 ± 34.35	128.23 ± 25.83	3.889	0.000
HAMD-17*	23.32 ± 5.69	–		–

MDD, major depressive disorder; BMI, Body mass index; IGF-1, Insulin-like growth factor 1; HAMD-17, Hamilton Depression Rating Scale.

*Data are expressed as the mean ± standard deviation.

### 3.2 Results of meta-analysis

**Figure 1:**
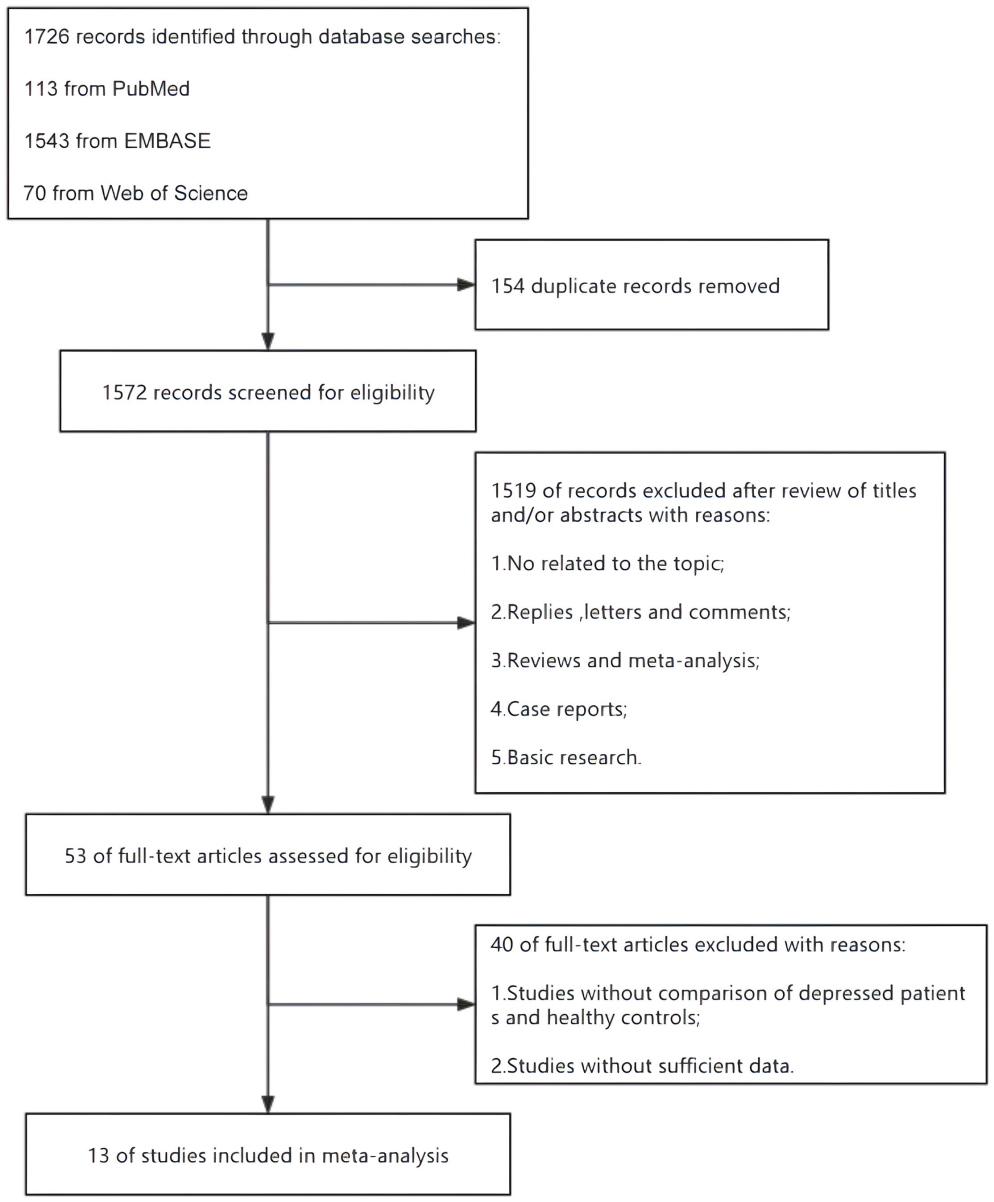
Flow diagram describing the process of study selection.

A flow diagram describing the study selection process is shown in **Figure 1**. A systematic literature search identified 1726 relevant articles in PubMed (n = 113), Embase (n = 1543) and Web of Science (n = 70). After deleting 154 duplicate papers, 1572 titles and abstracts were reviewed, of which 13 full-text articles were included in the meta-analysis (**Table 2**). Among them, 12 were case-control studies (15–20, 22–27), and 1 was a cross-sectional study with a group of healthy controls (21).

**Table 2:** The clinical characteristics of the included studies.

Authors (year)	Race	Study design	Sample source	Sample size (case/control)	% Female (case/control)	Mean age (case/control)	Depression rating instrument	Antidepressant use	*Correlation coefficient	NOS
Arinami.H(2023) (15)	Asian	case-control study	serum	129/71	46.51/46.48	40.5 ± 12.8/41.4 ± 9.3	HAMD-17	single	positive correlation	8
Suzuki.Y(2023) (16)	Asian	case-control study	serum	120/99	45.83/44.44	40.3 ± 12.8/40.8 ± 9.3	HAMD-17	mixed	NA	8
Arinami.H(2021) (17)	Asian	case-control study	serum	54/37	0.00/0.00	42.4 ± 14.7/39.4 ± 7.0	HAMD	mixed	absence of correlation	6
Troyan.A.S(2020) (18)	European	case-control study	serum	41/32	65.85/62.5	36.4 ± 12.8/38.0 ± 12.2	MADRS,CGI	no	positive correlation	7
Levada1.O.A(2020) (19)	European	case-control study	serum	78/47	61.54/57.45	38.2 ± 11.9/37.8 ± 12.3	MADRS,CGI	no	positive correlation	6
Rosso.G(2016) (20)	European	case-control study	serum	37/43	78.38/65.12	42.4 ± 11.9/42.3 ± 11.3	HAMD-17,CGI	no	absence of correlation	7
Bot.M(2016) (21)	European	cross-sectional study	plasma	2139/602	66.90/61.46	41.9 ± 12.6/41.0 ± 14.6	IDS	mixed	positive correlation	5
Kopczak.A(2014) (22)	European	case-control study	serum	78/96	44.87/43.75	48.6 ± 13.9/48.1 ± 13.7	HAMD-21	mixed	positive correlation	6
li(2013) (23)	Asian	case-control study	serum	15/12	0.00/0.00	32.3 ± 7.7/31.2 ± 10.2	MADRS	no	NA	6
Franza.B(1999) (24)	American	case-control study	serum	19/16	100.00/100.00	34.7 ± 8.8/36.1 ± 6.6	HAMD-17	mixed	NA	7
Deuschle.M(1997) (25)	European	case-control study	plasma	24/33	45.83/33.33	47.2 ± 16.4/51.4 ± 19.2	HAMD-21	no	NA	5
Michelson.D(1996) (26)	American	case-control study	serum	24/24	100.00/100.00	41.0 ± 8.0/41.0 ± 7.0	HAMD-17	mixed	NA	6
Lesch, K. P(1988) (27)	European	case-control study	plasma	34/34	67.65/67.65	48.2 ± 12.2/44.7 ± 11.9	HAMD-21	no	absence of correlation	5

HAMD, Hamilton Depression Scale; MADRS, Montgomery-Asberg Depression Scale; CGI-S, Clinical Global Impression Severity; IDS, 30-item self-reported Inventory of depressive symptomatology; NOS, Newcastle-Ottawa Scale; NA, not available.

*Correlation coefficient between IGF-1 level and depression severity.

**Figure 2:**
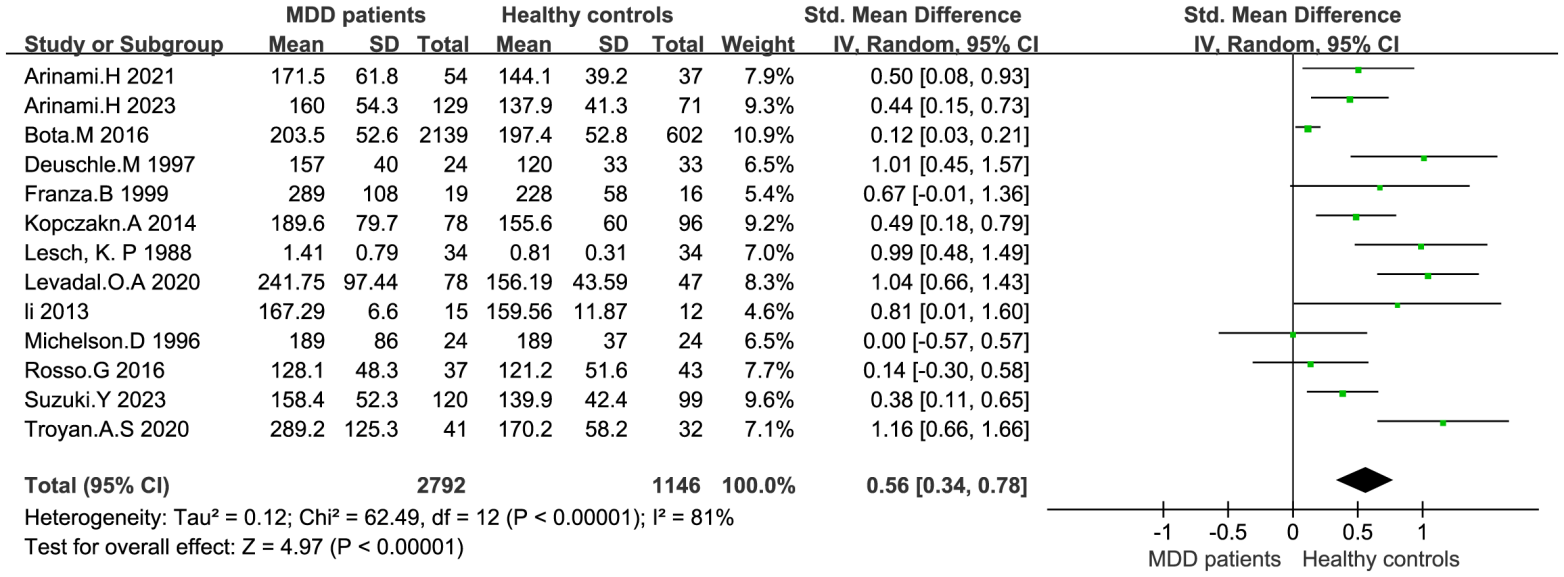
Forest plot of IGF-1.

**Figure 3:**
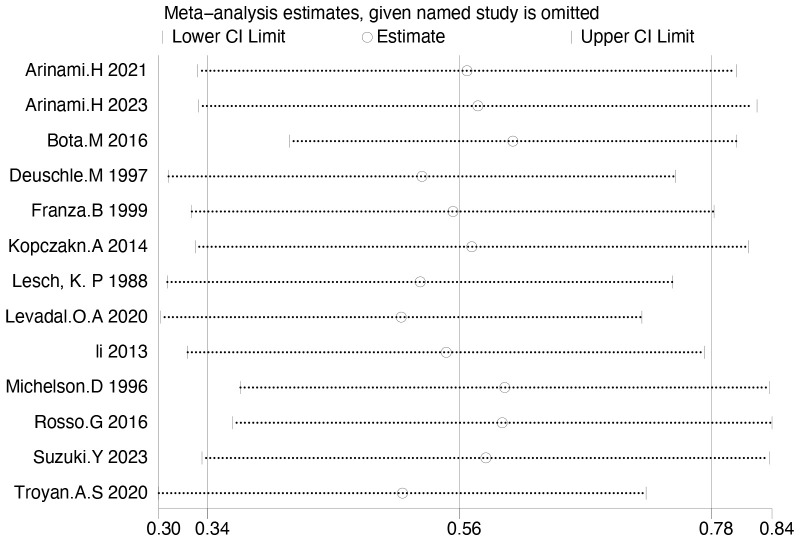
Sensitivity analysis of IGF-1.

**Figure 4:**
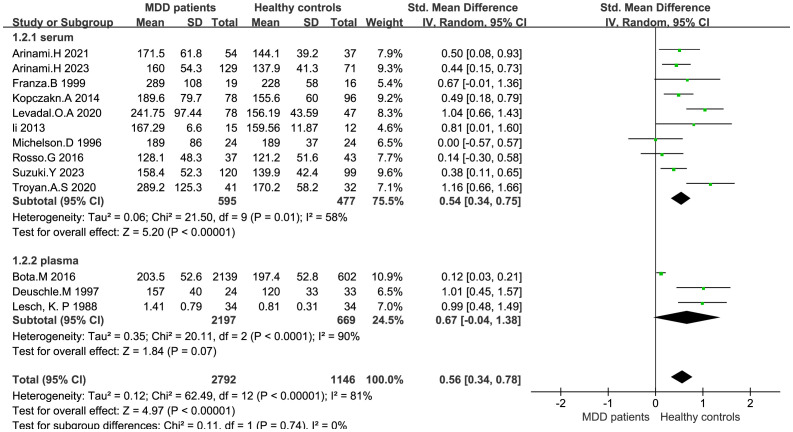
Forest plot of subgroup of sample source.

IGF-1 levels were compared between depressed patients (n=2792) and healthy controls (n=1146) from 13 studies. Combined results suggest that IGF-1 levels are significantly increased in MDD patients compared to healthy controls (SMD: 0.56; 95%CI: 0.34, 0.78; *p*<0.00001), with high heterogeneity (I^2^ = 81%; *p*<0.00001) (**Figure 2**). Sensitivity analyses indicated that the statistical significance of the pooled SMD did not change after excluding any single study (**Figure 3**). No significant publication bias was found by Egger’s test (*p*=0.928). No significant confounding factors were found to explain the high level of heterogeneity using β and subgroup analyses; however, subgroup analyses detected significant differences in IGF-1 levels between MDD patients and healthy controls only in serum samples, and studies measuring serum samples had lower heterogeneity than studies measuring plasma. (**Figure 4**).

## 4 Discussion

Results of the case-control study demonstrated that serum IGF-1 levels are significantly increased in patients with first-episode and drug-naïve depression compared with healthy controls independent of gender, age and severity of depression. The meta-analysis further demonstrated that IGF-1 levels in MDD patients is significantly higher than in healthy controls. These findings provide the latest evidence on serum IGF-1 levels in patients with first-episode and drug-naïve MDD compared with healthy controls, and supports existing meta-analyses demonstrating increased IGF-1 levels in MDD patients (4, 28–30).

Prior research has suggested that reduced IGF-1 expression in the brain disrupts neuroplasticity and promotes inflammatory pathways, leading to morphological deterioration of brain regions responsible for emotional and cognitive processes. Therefore, the increase in systemic IGF-1 levels observed in patients with mood disorders may be a compensatory mechanism that enhances the hypothalamic-pituitary-growth promotion axis in response to inadequate brain IGF-1 concentrations (11). Indeed, elevated peripheral IGF-1 levels suggest a reduced central effect of IGF-1 as an interacting mechanism. Studies in patients with affective disorders suggest that elevated IGF-1 levels may be related to its compensatory neuroprotective and angiogenic effects, and these proteins may be involved in the pathophysiological process of affective disorders (31). The increase of peripheral IGF-1 levels in patients with affective disorders may be related to the decline of cognitive function in such patients (32), although these findings remain inconclusive.

The present study investigated the correlation between age and IGF-1 levels in MDD patients and found no significant correlation between age and IGF-1 levels. This differs from some other results, which may be related to the age range of MDD patients that we included. According to preclinical results, short-term intervention of central IGF-1 can help reduce depression-like behavior in aged mice, and researchers have also shown that women over 90 years old have lower IGF-1 levels. (33, 34) However, reduced brain and peripheral IGF-1 expression in the elderly may be directly related to emotional and neuropsychological symptoms resulting from impaired mood and cognitive processing (11). In our study, only 18–65 years patients with first-episode and drug-naïve were included, with a mean age of 35.48 ± 11.77 years old. Age stratification was limited, and older people were not studied.” The different trends shown for IGF-1 in different age groups may indicate different mechanisms of action of neurotrophins in these populations, and more complete sample populations are needed for comparison.

Although there was no correlation between IGF-1 levels and the severity of depression in the current study, other studies report a positive correlation between these two factors (15, 21, 35). The present study points to a complex relationship between IGF-1 and depression; high and low levels of IGF-1 increased the prevalence of MDD in different phases of investigation, and mid concentrations decreased the probability of incident minor depression (29). In a study of older adults, decreased plasma IGF-1 levels were associated with decreased cognitive function and a higher prevalence of depressive symptoms (31). In the current study, the age distribution of the sample was not adequately differentiated due to sample size limitations, although a study on IGF-1 concentration and depression degree in different age groups may help explain this phenomenon. The diagnostic criteria for the degree of depression is also a factor to consider. Unlike the study by ROSSO et al (20), the present study collected serum samples from first-episode, drug-naïve patients with depression and completely excluded any possible effect of antidepressant use on IGF-1 levels. Factors that may have interfered with the measurement of IGF-1 levels were minimized, although the question of whether discontinuation of antidepressant therapy has a profound effect on IGF-1 levels remains unclear.

No gender differences between sample concentrations were found in the study, which differs from earlier studies reporting differences due to changes in sex hormones or fluctuations in growth hormone and IGF-1 binding proteins (29, 30, 36). The age of our study population was mostly young and middle-aged, which may be one of the factors affecting these results.

Meta-analyses demonstrated that IGF-1 levels in patients with depression were increased in serum samples but not plasma samples. Shi et al. reported the opposite (4), although the differences in sample size between the two studies may be an important factor.

There are several limitations to this study: (1) Although we strictly controlled the enrollment criteria and collected patients with first-episode, drug-naïve depression, there was no restriction on the duration of onset, which may have affected the accuracy of results. (2) Heterogeneity among studies was found in the meta-analysis, and although detailed meta-regression and subgroup analyses were used to exclude confounding factors, no potential sources were found to explain this heterogeneity. The inconsistency of population samples may contribute to the heterogeneity of studies. Therefore, the current results should be interpreted with caution. (3) Finally, the inclusion of cognition level in the study may have provided additional evidence for the interpretation of the results, and future studies may take this variable into account.

## 5 Conclusion

Patients with first-episode and drug-naïve depression have higher IGF-1 levels, but the exclusion of confounding factors in studies of IGF-1 as it relates to depressive disorders must be taken into consideration strictly, and additional research is needed to fully understand the critical role of IGF-1 in depression.

## Data availability statement

The raw data supporting the conclusions of this article will be made available by the authors, without undue reservation.

## Ethics statement

The studies involving humans were approved by Ethics Committee of the Fourth People’s Hospital of Wuhu. The studies were conducted in accordance with the local legislation and institutional requirements. The participants provided their written informed consent to participate in this study.

## Author contributions

XQ: Writing – original draft, Formal analysis. JY: Writing – original draft, Data curation. ZZ: Writing – original draft, Data curation. LX: Writing – original draft, Data curation. WZ: Writing – review & editing, Formal analysis. EZ: Writing – review & editing, Project administration. LW: Writing – review & editing.
