# Supplementary material for: Association between IGF-1 levels and MDD: a case-control and meta-analysis
Source: Front Psychiatry. 2024 Jun 11;15:1396938. doi: 10.3389/fpsyt.2024.1396938 (PMC11196772; doi:10.3389/fpsyt.2024.1396938)
Supplement: Supplementary file 1 [file DataSheet_1.zip › Supplementary Material 2.DOCX]

**Supplementary Table2** Search Strategy

| Search | Query |
| --- | --- |
| #1 | "Depressive Disorder"[MeSH Terms] OR ("Depressive Disorder"[MeSH Terms] OR ("depressive"[All Fields] AND "disorder"[All Fields]) OR "Depressive Disorder"[All Fields] OR ("depressive"[All Fields] AND "disorders"[All Fields]) OR "depressive disorders"[All Fields] OR ("Depressive Disorder"[MeSH Terms] OR ("depressive"[All Fields] AND "disorder"[All Fields]) OR "Depressive Disorder"[All Fields] OR ("disorder"[All Fields] AND "depressive"[All Fields]) OR "disorder depressive"[All Fields]) OR ("Depressive Disorder"[MeSH Terms] OR ("depressive"[All Fields] AND "disorder"[All Fields]) OR "Depressive Disorder"[All Fields] OR ("disorders"[All Fields] AND "depressive"[All Fields]) OR "disorders depressive"[All Fields]) OR ("Depressive Disorder"[MeSH Terms] OR ("depressive"[All Fields] AND "disorder"[All Fields]) OR "Depressive Disorder"[All Fields] OR ("neurosis"[All Fields] AND "depressive"[All Fields]) OR "neurosis depressive"[All Fields]) OR ("Depressive Disorder"[MeSH Terms] OR ("depressive"[All Fields] AND "disorder"[All Fields]) OR "Depressive Disorder"[All Fields] OR ("depressive"[All Fields] AND "neuroses"[All Fields]) OR "depressive neuroses"[All Fields]) OR ("Depressive Disorder"[MeSH Terms] OR ("depressive"[All Fields] AND "disorder"[All Fields]) OR "Depressive Disorder"[All Fields] OR ("depressive"[All Fields] AND "neurosis"[All Fields]) OR "depressive neurosis"[All Fields]) OR ("Depressive Disorder"[MeSH Terms] OR ("depressive"[All Fields] AND "disorder"[All Fields]) OR "Depressive Disorder"[All Fields] OR ("neuroses"[All Fields] AND "depressive"[All Fields]) OR "neuroses depressive"[All Fields]) OR ("Depressive Disorder"[MeSH Terms] OR ("depressive"[All Fields] AND "disorder"[All Fields]) OR "Depressive Disorder"[All Fields] OR ("neuroses"[All Fields] AND "depressive"[All Fields]) OR "neuroses depressive"[All Fields]) OR ("Depressive Disorder"[MeSH Terms] OR ("depressive"[All Fields] AND "disorder"[All Fields]) OR "Depressive Disorder"[All Fields] OR ("depressions"[All Fields] AND "endogenous"[All Fields]) OR "depressions endogenous"[All Fields]) OR ("Depressive Disorder"[MeSH Terms] OR ("depressive"[All Fields] AND "disorder"[All Fields]) OR "Depressive Disorder"[All Fields] OR ("endogenous"[All Fields] AND "depression"[All Fields]) OR "endogenous depression"[All Fields]) OR ("Depressive Disorder"[MeSH Terms] OR ("depressive"[All Fields] AND "disorder"[All Fields]) OR "Depressive Disorder"[All Fields] OR ("endogenous"[All Fields] AND "depressions"[All Fields]) OR "endogenous depressions"[All Fields]) OR ("Depressive Disorder"[MeSH Terms] OR ("depressive"[All Fields] AND "disorder"[All Fields]) OR "Depressive Disorder"[All Fields] OR ("depressive"[All Fields] AND "syndrome"[All Fields]) OR "depressive syndrome"[All Fields]) OR ("Depressive Disorder"[MeSH Terms] OR ("depressive"[All Fields] AND "disorder"[All Fields]) OR "Depressive Disorder"[All Fields] OR ("depressive"[All Fields] AND "syndromes"[All Fields]) OR "depressive syndromes"[All Fields]) OR ("Depressive Disorder"[MeSH Terms] OR ("depressive"[All Fields] AND "disorder"[All Fields]) OR "Depressive Disorder"[All Fields] OR ("syndrome"[All Fields] AND "depressive"[All Fields]) OR "syndrome depressive"[All Fields]) OR ("Depressive Disorder"[MeSH Terms] OR ("depressive"[All Fields] AND "disorder"[All Fields]) OR "Depressive Disorder"[All Fields] OR ("syndromes"[All Fields] AND "depressive"[All Fields]) OR "syndromes depressive"[All Fields]) OR ("Depressive Disorder"[MeSH Terms] OR ("depressive"[All Fields] AND "disorder"[All Fields]) OR "Depressive Disorder"[All Fields] OR ("depression"[All Fields] AND "neurotic"[All Fields]) OR "depression neurotic"[All Fields]) OR ("Depressive Disorder"[MeSH Terms] OR ("depressive"[All Fields] AND "disorder"[All Fields]) OR "Depressive Disorder"[All Fields] OR ("depressions"[All Fields] AND "neurotic"[All Fields])) OR ("Depressive Disorder"[MeSH Terms] OR ("depressive"[All Fields] AND "disorder"[All Fields]) OR "Depressive Disorder"[All Fields] OR ("neurotic"[All Fields] AND "depression"[All Fields]) OR "neurotic depression"[All Fields]) OR ("Depressive Disorder"[MeSH Terms] OR ("depressive"[All Fields] AND "disorder"[All Fields]) OR "Depressive Disorder"[All Fields] OR ("neurotic"[All Fields] AND "depressions"[All Fields]) OR "neurotic depressions"[All Fields]) OR ("Depressive Disorder"[MeSH Terms] OR ("depressive"[All Fields] AND "disorder"[All Fields]) OR "Depressive Disorder"[All Fields] OR "melancholia"[All Fields] OR "melancholias"[All Fields]) OR ("Depressive Disorder"[MeSH Terms] OR ("depressive"[All Fields] AND "disorder"[All Fields]) OR "Depressive Disorder"[All Fields] OR "melancholia"[All Fields] OR "melancholias"[All Fields]) OR ("Depressive Disorder"[MeSH Terms] OR ("depressive"[All Fields] AND "disorder"[All Fields]) OR "Depressive Disorder"[All Fields] OR ("unipolar"[All Fields] AND "depression"[All Fields]) OR "unipolar depression"[All Fields]) OR ("Depressive Disorder"[MeSH Terms] OR ("depressive"[All Fields] AND "disorder"[All Fields]) OR "Depressive Disorder"[All Fields] OR ("depression"[All Fields] AND "unipolar"[All Fields]) OR "depression unipolar"[All Fields]) OR ("Depressive Disorder"[MeSH Terms] OR ("depressive"[All Fields] AND "disorder"[All Fields]) OR "Depressive Disorder"[All Fields] OR ("depressions"[All Fields] AND "unipolar"[All Fields]) OR "depressions unipolar"[All Fields]) OR ("Depressive Disorder"[MeSH Terms] OR ("depressive"[All Fields] AND "disorder"[All Fields]) OR "Depressive Disorder"[All Fields] OR ("unipolar"[All Fields] AND "depressions"[All Fields]) OR "unipolar depressions"[All Fields])) |
| #2 | "Insulin-Like Growth Factor I"[MeSH Terms] OR "Insulin-Like Growth Factor I"[MeSH Terms] OR "Insulin-Like Growth Factor I"[All Fields] OR "insulin like somatomedin peptide i"[All Fields] OR "Insulin-Like Growth Factor I"[MeSH Terms] OR "Insulin-Like Growth Factor I"[All Fields] OR "insulin like somatomedin peptide i"[All Fields] OR "Insulin-Like Growth Factor I"[MeSH Terms] OR "Insulin-Like Growth Factor I"[All Fields] OR "somatomedin c"[All Fields] OR "Insulin-Like Growth Factor I"[MeSH Terms] OR "Insulin-Like Growth Factor I"[All Fields] OR "igf i smc"[All Fields] OR "Insulin-Like Growth Factor I"[MeSH Terms] OR "Insulin-Like Growth Factor I"[All Fields] OR "igf 1"[All Fields] OR "Insulin-Like Growth Factor I"[MeSH Terms] OR "Insulin-Like Growth Factor I"[All Fields] OR "igf i"[All Fields] OR "Insulin-Like Growth Factor I"[MeSH Terms] OR "Insulin-Like Growth Factor I"[All Fields] OR "Insulin-Like Growth Factor I"[All Fields] |
| #3 | #1 AND #2 |
