# Supplementary material for: Association between IGF-1 levels and MDD: a case-control and meta-analysis
Source: Front Psychiatry. 2024 Jun 11;15:1396938. doi: 10.3389/fpsyt.2024.1396938 (PMC11196772; doi:10.3389/fpsyt.2024.1396938)
Supplement: Supplementary file 1 [file DataSheet_1.zip › Supplementary Material 3.DOCX]

**Supplementary 3** Normality tests for continuous variables in case-control study

**Table 1** Skewness coefficient and kurtosis coefficient：

|  | Skewness |  | Kurtosis |  |
| --- | --- | --- | --- | --- |
|  | MDD | Control | MDD | Control |
| Age | 0.643 | 0.597 | -0.685 | -1.010 |
| BMI | 0.580 | 0.919 | 0.083 | 4.275 |
| IGF-1 | -0.277 | 0.951 | -0.638 | 0.988 |

**Figure 1** Age distribution Histograms of MDD patients.


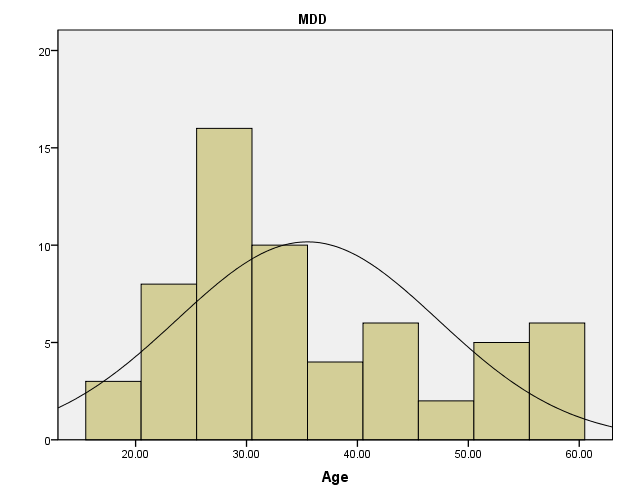


**Figure 2** Age distribution Histograms of controls.


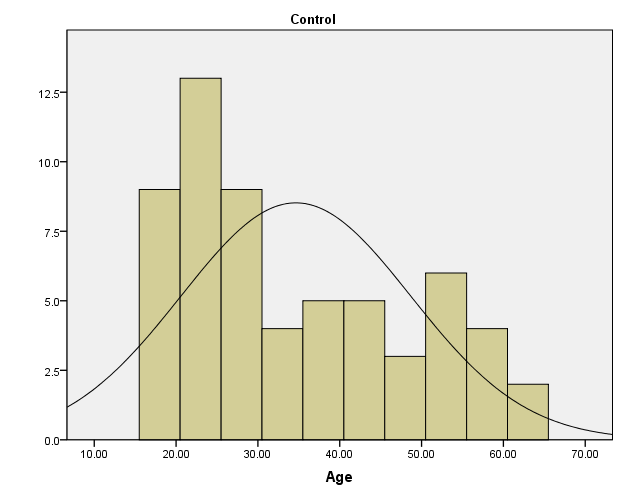


**Figure 3** Q-Q plot of age distribution of MDD patients.


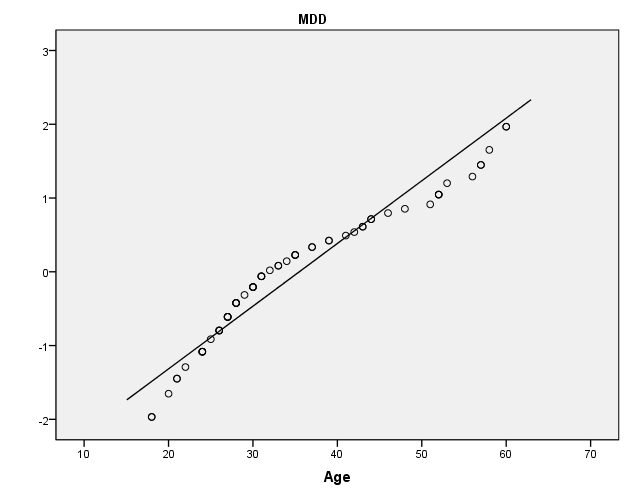


**Figure 4** Q-Q plot of age distribution of controls.


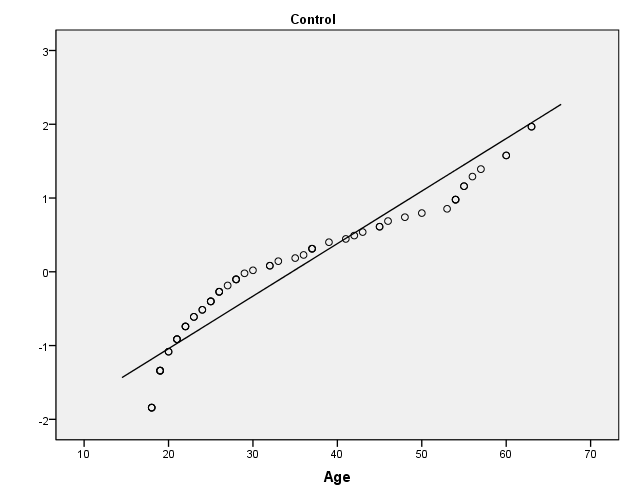


**Figure 5** BMI distribution Histograms of MDD patients


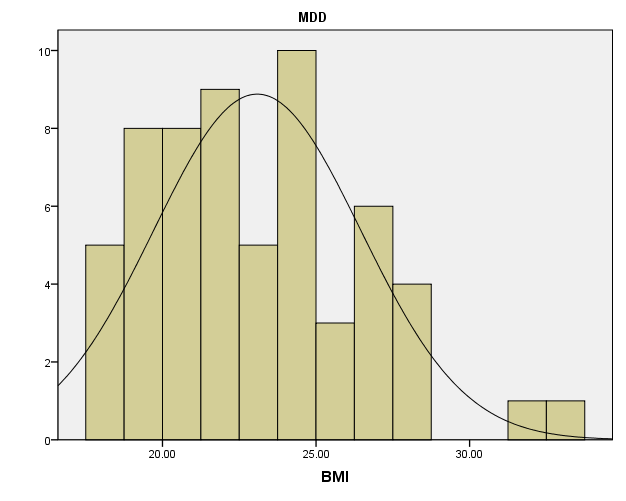


**Figure 6** BMI distribution Histograms of controls


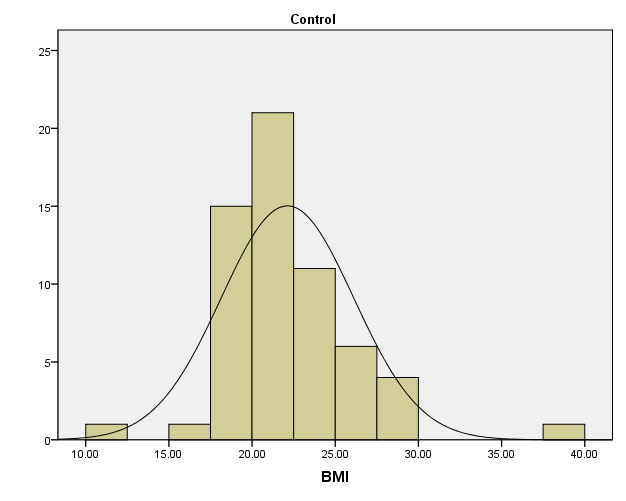


**Figure 7** Q-Q plot of BMI distribution of MDD patients.


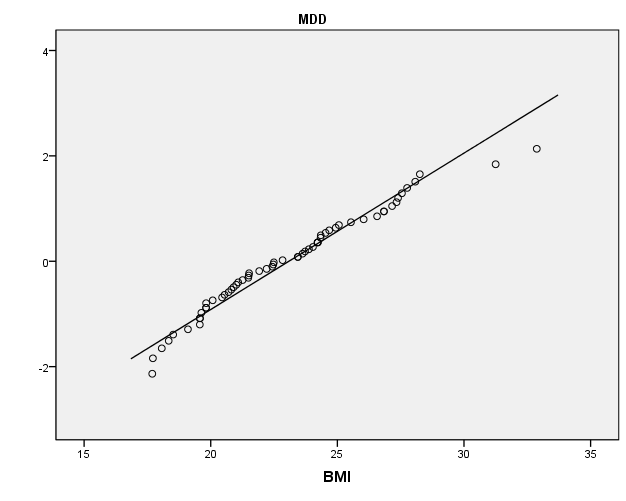


**Figure 8** Q-Q plot of BMI distribution of controls.


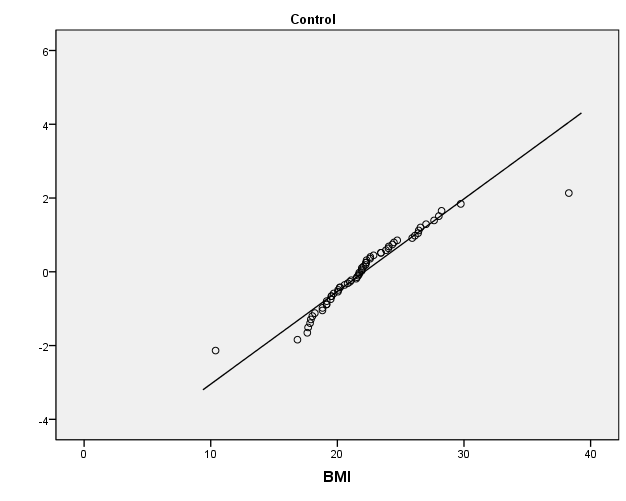


**Figure 9** IGF-1 level distribution Histograms of MDD patients


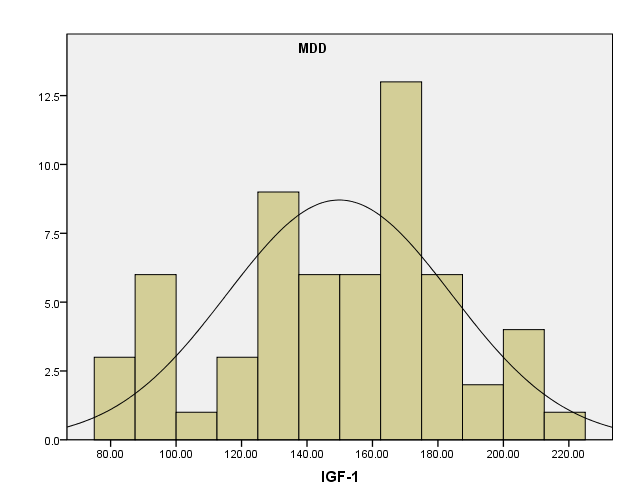


**Figure 10** IGF-1 level distribution Histograms of controls


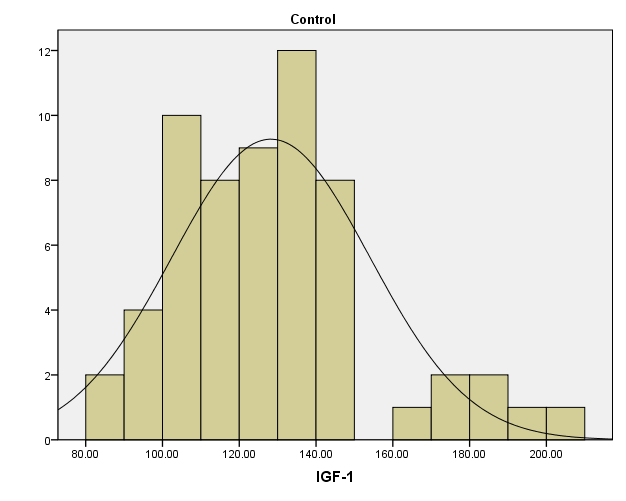


**Figure 11** Q-Q plot of IGF-1 level distribution of MDD patients.


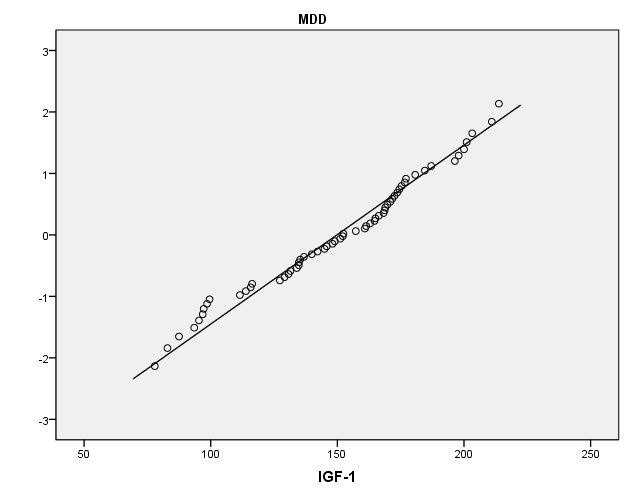


**Figure 12** Q-Q plot of IGF-1 level distribution of controls.


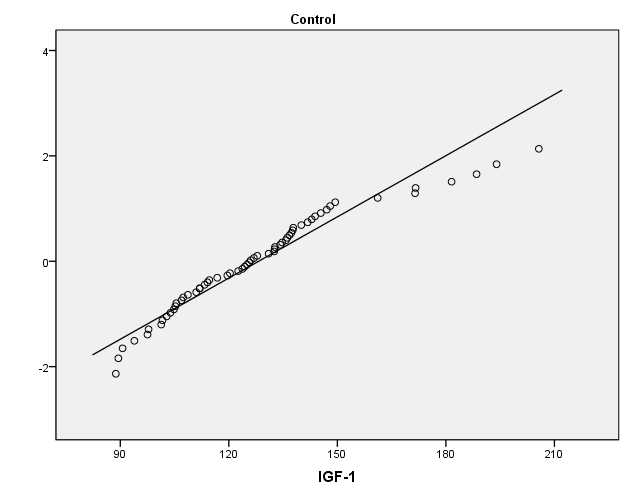


**Table 2** Kolmogorov-Smirnov assumption test.

| Kolmogorov-Smirnov |  | D-value | df | *p*-value |
| --- | --- | --- | --- | --- |
| Age | MDD | 0.148 | 60 | 0.002 |
|  | Control | 0.165 | 60 | 0.000 |
| BMI | MDD | 0.096 | 60 | 0.200 |
|  | Control | 0.119 | 60 | 0.033 |
| IGF-1 | MDD | 0.093 | 60 | 0.200 |
|  | Control | 0.105 | 60 | 0.097 |
